# TIPE-mediated up-regulation of MMP-9 promotes colorectal cancer invasion and metastasis through MKK-3/p38/NF-κB pro-oncogenic signaling pathway

**DOI:** 10.1038/s41392-020-00276-7

**Published:** 2020-08-25

**Authors:** Huiyu Chen, Yuhan Ye, Yan Yang, Mengya Zhong, Lei Gu, Zhaopu Han, Jinhua Qiu, Zhongchen Liu, Xingfeng Qiu, Guohong Zhuang

**Affiliations:** 1 Cancer Research Center, School of Medicine, Xiamen University, Xiamen, Fujian China; 2 Department of Pathology, Zhongshan Hospital Affiliated to Xiamen University, Xiamen, Fujian China; 3 Department of General Surgery, Shanghai 10th People’s Hospital Affiliated to Tongji University, Shanghai, China; 4 Department of Gastrointestinal Surgery, Zhongshan Hospital Affiliated to Xiamen University, Xiamen, Fujian China; 5 Organ Transplantation Institute of Xiamen University, Fujian Provincial Key Laboratory of Organ and Tissue Regeneration, School of Medicine, Xiamen University, Xiamen, Fujian China

**Keywords:** Gastrointestinal cancer, Tumour biomarkers

**Dear Editor,**

Colorectal cancer (CRC) is the third most common malignant tumor in human, ranking third in cancer-related mortality.^1^ Most colon cancer patients die of metastasis.^2^

Intriguingly, the deregulation of tumor necrosis factor α‑induced protein 8 (TIPE) has been shown to play a vital regulatory role in tumor cell growth, proliferation, invasion, and metastasis.^3^ TIPE is a kind of cytoplasmic protein of 23 kDa. A large number of studies have shown that TIPE is closely related to the development of colon cancer.^4^ In addition, decreased expression of TIPE was linked to down-regulation of matrix metallopeptidase-1 (MMP-1), MMP-9, and vascular endothelial growth factor receptor-2 in breast cancer.^5^ In addition, MMP-9 plays a vital role in the degradation and destruction of extracellular matrix components, and tumor cell invasion and metastasis. However, their functional role during CRC metastasis is still elusive. Thus, we hypothesized that TIPE could promote CRC metastasis by regulating the expression of MMP-9. To address this issue, we analyzed the expression of TIPE and MMP-9 in CRC patients’ tissue samples and human CRC cell lines. The results showed that TIPE and MMP-9 expression were significantly up-regulated in CRC tissues compared to adjacent tissues (Supplementary Fig. S1a–c) and the results showed that MMP-9 was positively correlated with TIPE (Supplementary Fig. S1d). Moreover, according to the clinical organization results, we found that TIPE is associated with lymph node metastasis (Supplementary Table 1). According to the expression level of TIPE in CRC cells (Supplementary Fig. S2a), we constructed a stable expression of TIPE-depleted in HCT116 and TIPE overexpression in SW480 cell lines (Supplementary Fig. S2b, c). To investigate whether TIPE and MMP-9 are positively correlated in cell lines, we have examined the endogenous expression of MMP-9 in stable cell lines. As shown Fig. 1a, the endogenous expression level of MMP-9 was decreased in TIPE-depleted HCT116 cells and increased TIPE-overexpressing SW480 cells. The results further indicate that TIPE might be involved in the regulation of CRC invasion as well as migration through up-regulation of MMP-9.

**Fig. 1:**
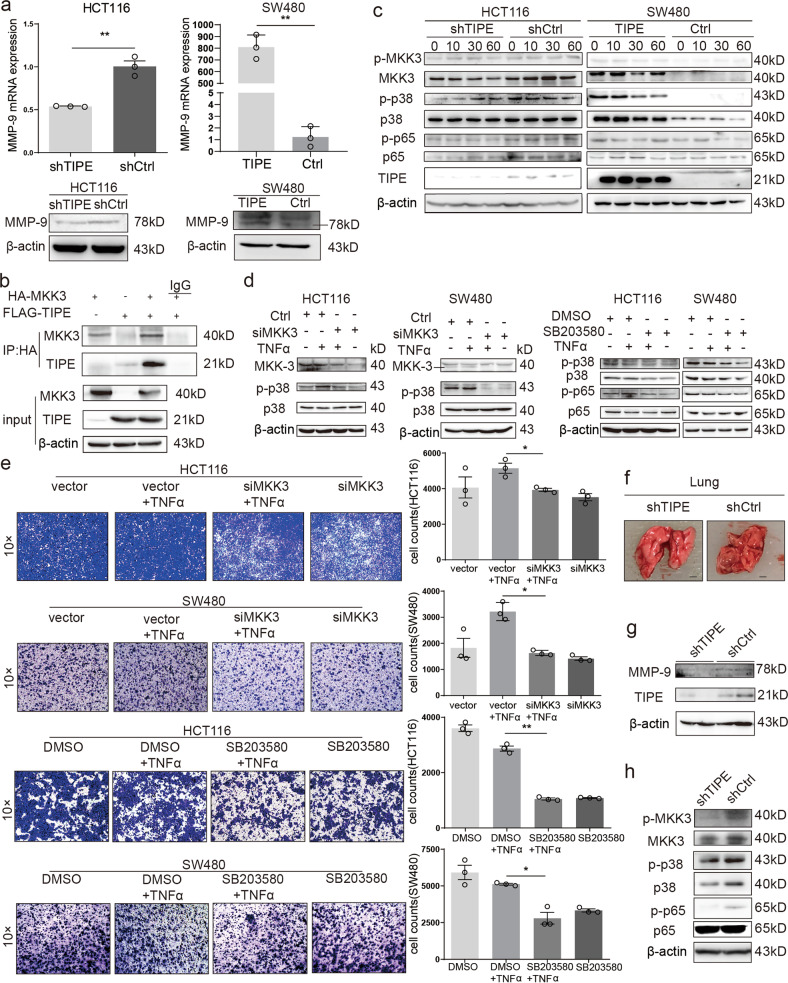
**a** qRT-PCR and Western blot detection of MMP-9 mRNA and protein expression in TIPE-depleted HCT116 cells and TIPE-overexpressing SW480 cells. **b** Complex formation between TIPE and MKK-3 in cells. HEK293T cells were co-transfected with the expression plasmid for Flag-TIPE and HA-MKK-3, then whole-cell lysates were analyzed by co-immunoprecipitation. **c** TNF-α-mediated alteration of the phosphorylation levels of MKK-3/p38/NF-κB as examined by Western blotting. TIPE-depleted HCT116 cells (left panels) and TIPE-overexpressed SW480 cells (right panels) were exposed to TNF-α. At the indicated time points after the treatment, whole-cell lysates were analyzed by the indicated antibodies. **d** The effect of MKK-3 knockdown and the inhibitor for p38 on the phosphorylation level of p38/NF-κB in TIPE-depleted HCT116 and TIPE-overexpressed SW480 cells. **e** siRNA-mediated silencing of MKK-3 or the addition of p38 inhibitor (SB203580) attenuated cell invasion. Graphical representation of the cell invasion of the Transwell experiment described in the data. **f** Anatomy diagram of mice lung metastases. Scale bar, 2 mm. **g** TIPE and MMP-9 expressions in lung metastases were examined by Western blotting. β-Actin was used as an internal reference. **h** The effect of TIPE silencing on the phosphorylation levels of MKK-3, p38, and p65 was examined by Western blotting. β-Actin was used as an internal reference. *p < 0.05; **p < 0.01; (mean ± s.e.m. in three separate experiments)

To clarify the precise molecular mechanisms by which TIPE could regulate MMP-9 expression, we use mass spectrometry (Supplementary Fig. S3a) and the results suggest that TIPE might interact with mitogen-activated protein kinase kinase 3 (MKK-3). Therefore, we carried out co-immunoprecipitation analysis to check the interaction between TIPE and MKK-3 (Fig. 1b).

We treated stable transfected cell lines with 10 ng/ml tumor necrosis factor-α. As shown in Fig. 1c, TIPE depletion in HCT116 cells down-regulated the phosphorylation (Ser-189) and the total expression of MKK-3. Consistent with these results, TIPE overexpression in SW480 cells induced the phosphorylation of MKK-3 and the expression level of total MKK-3 was also elevated. We then examined the changes of MKK-3 downstream targets and found that, similar to MKK-3, the expression and phosphorylation levels of p38 at Thr-180/Tyr-182/NF-κB (nuclear factor-κB) at Ser-536 were decreased in TIPE-knockdown HCT116 cells and increased TIPE-overexpressing SW480 cells. Therefore, it is possible that TIPE might regulate MMP-9 expression through the phosphorylation of MKK-3/p38/NF-κB.

To further verify these results, we added siMKK3 or SB203580 to inhibit the expression of MKK-3 and p38. As seen in the left panels of Fig. 1d, knockdown of MKK-3 resulted in a significant reduction of the phosphorylation level of p38, respectively. As shown in the right panels of Fig. 1d, the phosphorylation level of NF-κB was reduced and elevated in TIPE-depleted HCT116 cells or SW480 cells overexpressing TIPE in response to SB203580, respectively. These results suggest that TIPE might regulate MMP-9 expression through the activation of MKK-3/p38/NF-κB pro-oncogenic pathway.

Based on the above observations, we sought to verify the functional significance of MKK-3/p38 in CRC. As shown in Fig. 1e, silencing of MKK-3 led to the reduction of their invasion ability. Then, we added the p38 inhibitor SB203580. As expected, the inhibition of p38 attenuated the invasion. These results indicate that both MKK-3 and p38 are implicated in CRC cell invasion, and TIPE might promote the migration and invasion of CRC cells via the activation of MKK-3/p38.

In light of our in vitro findings, we asked the possible effect of TIPE on HCT116 cell-induced metastasis in vivo. As clearly shown in Fig. 1f and Supplementary Fig. S5a, Hematoxylin and eosin results showed that cancer cells metastasized in the lungs of the control group, but no metastasis was observed in the TIPE-knockdown group, which indicates that knockdown of TIPE significantly prohibits tumor metastasis. In addition, we checked the expression of TIPE and MMP-9 in lung tissues of TIPE-knockdown group by Western blotting. As shown in Fig. 1g, the expression levels of TIPE and MMP-9 in TIPE-knockdown group were reduced. Western blotting demonstrated that MKK-3/p38/NF-κB phosphorylation levels are lower in TIPE*-*knockdown group than those in the control group (Fig. 1h), and the real-time quantitative PCR has shown that the expression of TIPE and MMP-9 is lower in TIPE*-*knockdown group than those in the control group (Supplementary Fig. S5b).These in vivo results were consistent with in vitro results. These observations indicate that knockdown of TIPE significantly inhibits tumorigenicity and metastasis in nude mice.

In conclusion, we verified a positive correlation between TIPE and MMP-9 and revealed that TIPE interact with MKK-3. Taken together, our current study strongly suggests that TIPE/MMP-9 regulatory axis participates in the metastasis of CRC through the MKK-3/p38/NF-κB pro-oncogenic pathway. Therefore, these gene products might be the potential molecular targets to develop a novel strategy to treat metastatic CRC patients.

## Supplementary information

SUPPLEMENTAL INFORMATION

Supplementary Table 1. The relationship between TIPE and clinicopathological factors of CRC patients

Supplementary Table 2
