# Supplementary material for: TIPE-mediated up-regulation of MMP-9 promotes colorectal cancer invasion and metastasis through MKK-3/p38/NF-κB pro-oncogenic signaling pathway
Source: Signal Transduct Target Ther. 2020 Aug 25;5:163. doi: 10.1038/s41392-020-00276-7 (PMC7447793; doi:10.1038/s41392-020-00276-7)
Supplement: Supplementary file 1 — SUPPLEMENTAL INFORMATION [file 41392_2020_276_MOESM1_ESM.docx]

**Supplementary Materials for**

**TIPE-mediated up-regulation of MMP-9 promotes colorectal cancer invasion and metastasis through MKK-3/p38/NF-κB pro-oncogenic signaling pathway**

Huiyu Chen, Yuhan Ye, Yan Yang, Mengya Zhong, Lei Gu, Zhaopu Han, Jinhua Qiu, Zhongchen Liu, Xingfeng Qiu, Guohong Zhuang

Contact: Guohong Zhuang, Email: zhgh@xmu.edu.cn; Zhongchen Liu, Email: 13860184888@163.com; Xingfeng Qiu, Email: Dr.qxf@xmu.edu.cn

**Supplementary information, Figures**

Fig.S1. TIPE and MMP-9 are highly expressed in CRC tissues.

Fig.S2. Construction of stable transfected cell lines.

Fig.S3. TIPE interacts with MKK-3.

Fig.S4. TIPE regulates MMP-9 expression through the phosphorylation of MKK-3/p38 /NF-κB.

Fig.S5. Knockdown of TIPE decreases MMP-9 expression level and prohibits CRC cell metastasis *in vivo.*

**Supplementary information, Materials and Methods**

**
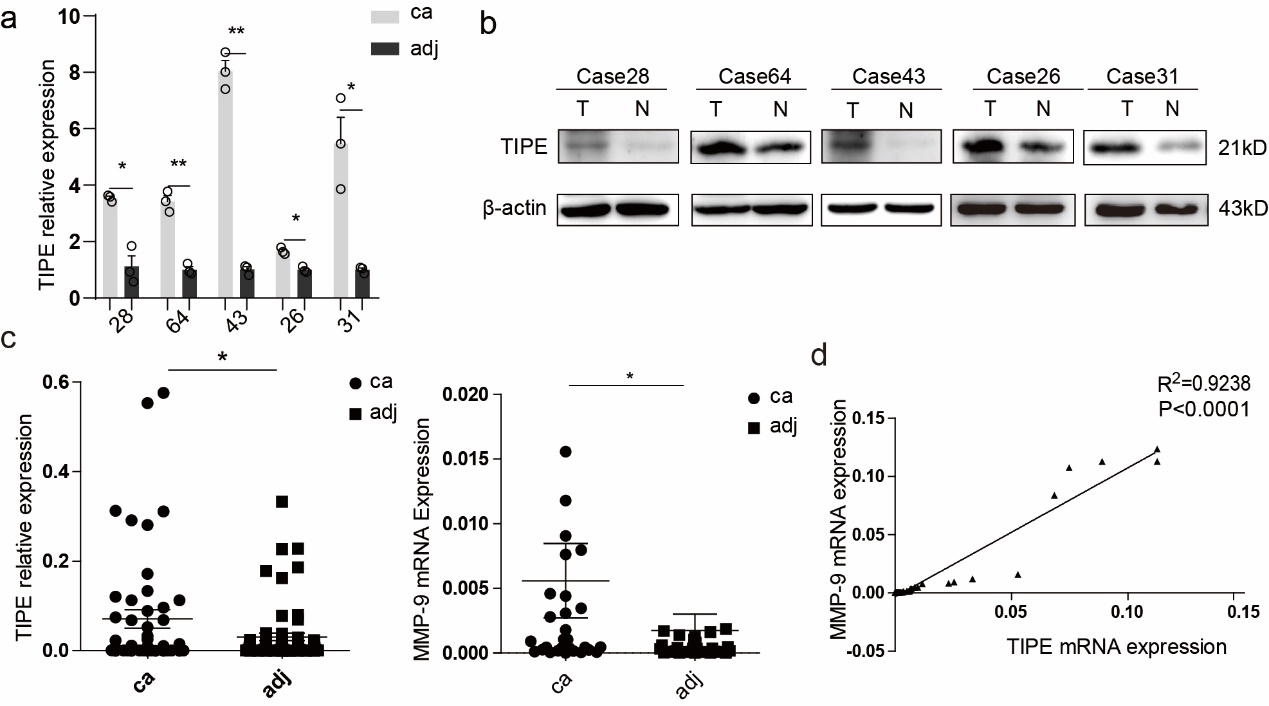
**

**Fig.S1.** TIPE and MMP-9 are highly expressed in CRC tissues

**a** mRNA expression level of TIPE in 5 pairs of CRC tissues and their corresponding adjacent ones was analyzed by qRT-PCR. The logarithmic scale of 2^-ΔΔCt^ was used to measure the fold change. β-actin was used as an internal reference. Ca, CRC tissues; adj, adjacent tissues. **b** TIPE protein expression level in 5 pairs of CRC tissues was examined by Western blotting. β-actin was used as a loading control. Case, CRC tissues; N, adjacent tissues; T, tumor tissues **c** mRNA expression level of TIPE in 30 pairs of CRC tissues and their corresponding adjacent ones was examined by qRT-PCR (left). mRNA expression level of MMP-9 in 30 pairs of CRC tissues and their corresponding adjacent ones was examined by qRT-PCR (right). **d** Correlation between mRNA expression levels of TIPE and MMP-9 in CRC tissue samples. * p <0.05; ** p <0.01（mean±s.e.m in three separate experiments）


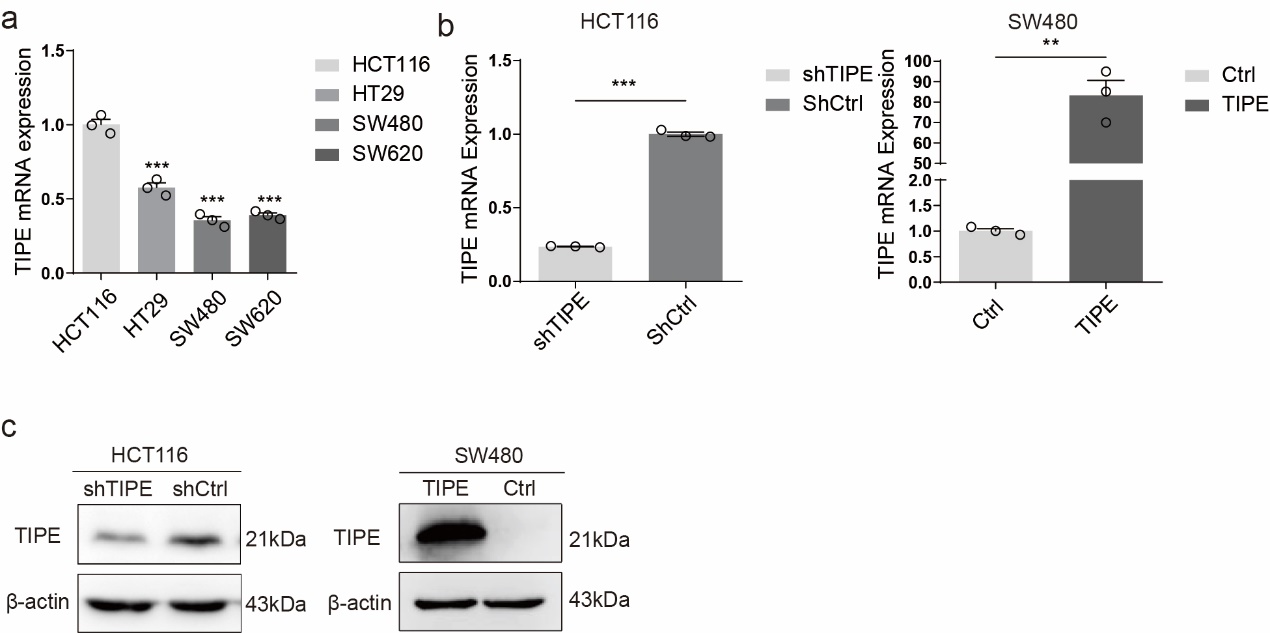


**Fig. S2.** Construction of stable transfected cell lines

**a** qRT-PCR was used to detect TIPE expression in CRC cells. The logarithmic scale of 2^-ΔΔCt^ was used to measure fold change. β-actin as internal reference. **b, c** forced expression and shRNA-mediated silencing of TIPE. The efficiency of TIPE overexpression and TIPE knockdown was checked by Western blotting and qRT-PCR, respectively. β-actin was used as an internal control and a loading control. ** p <0.01; *** p <0.001（mean±s.e.m in three separate experiments）


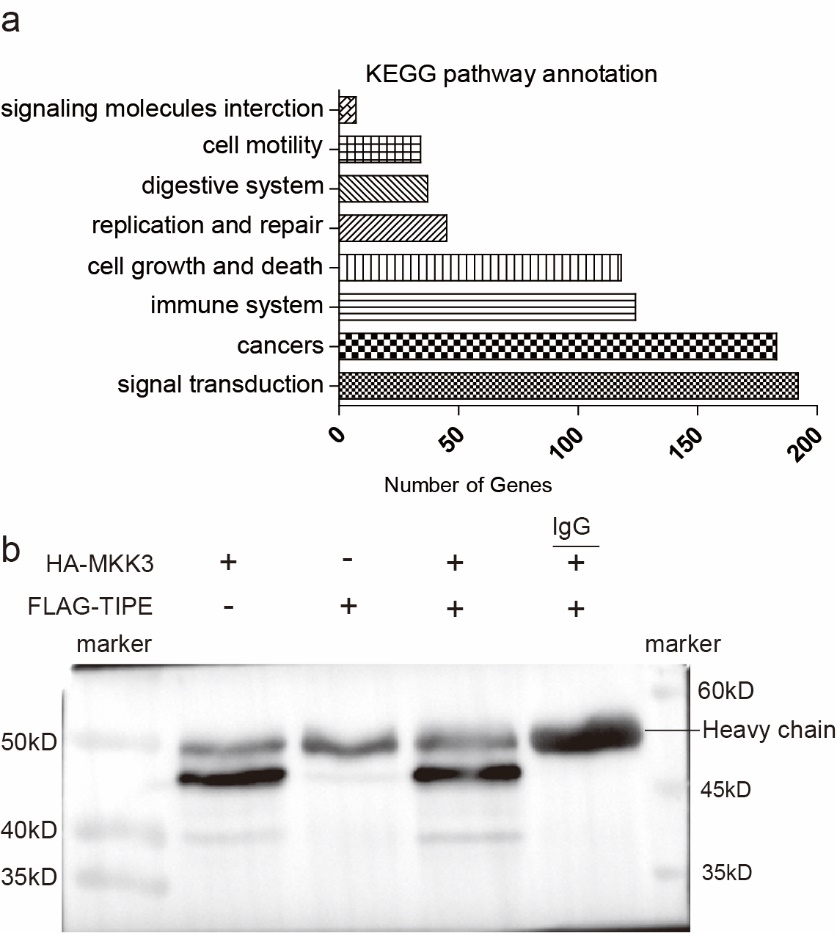


**Fig. S3**. TIPE interacts with MKK-3

**a** Mass spectrum results. **b** IP results with protein marker.


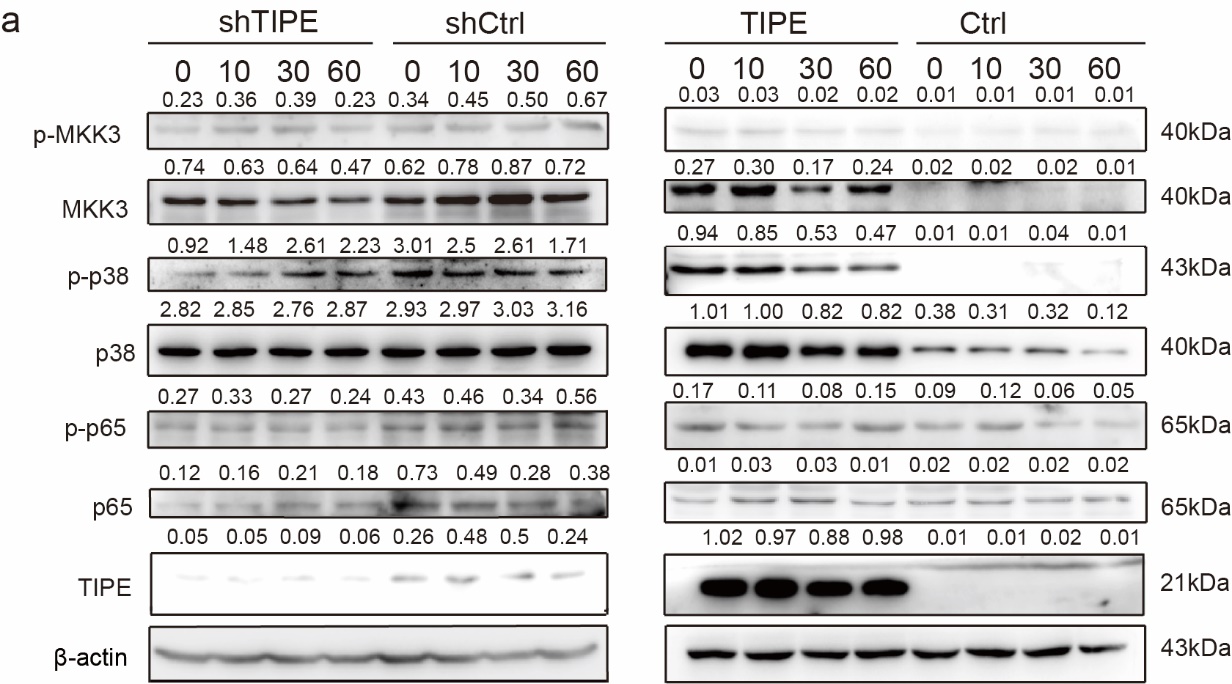


**Fig.S4**. TIPE regulates MMP-9 expression through the phosphorylation of MKK-3/p38 /NF-κB.

**a** Quantitative results of TNFα-mediated alteration of the phosphorylation levels of MKK-3/p38/NF-κB as examined by Western blotting.


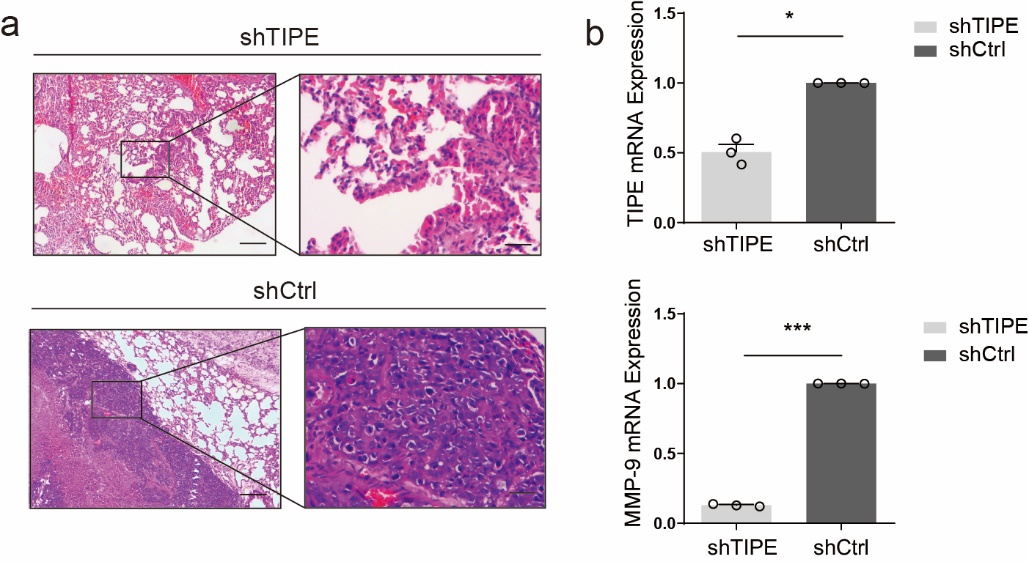


**Fig.S5.** Knockdown of TIPE decreases MMP-9 expression level and prohibits CRC cell metastasis in vivo

**a** Mice lung metastases were checked by H&Estaining.Scalebar,100μm (left),20μm(right). **b** TIPE and MMP-9 expression in lung metastases were examined by qRT-PCR. The logarithmic scale of 2^-ΔΔCt^ was used to measure the fold change. β-actin was used as an internal reference. * p <0.05; *** p <0.001. （mean±s.e.m in three separate experiments）


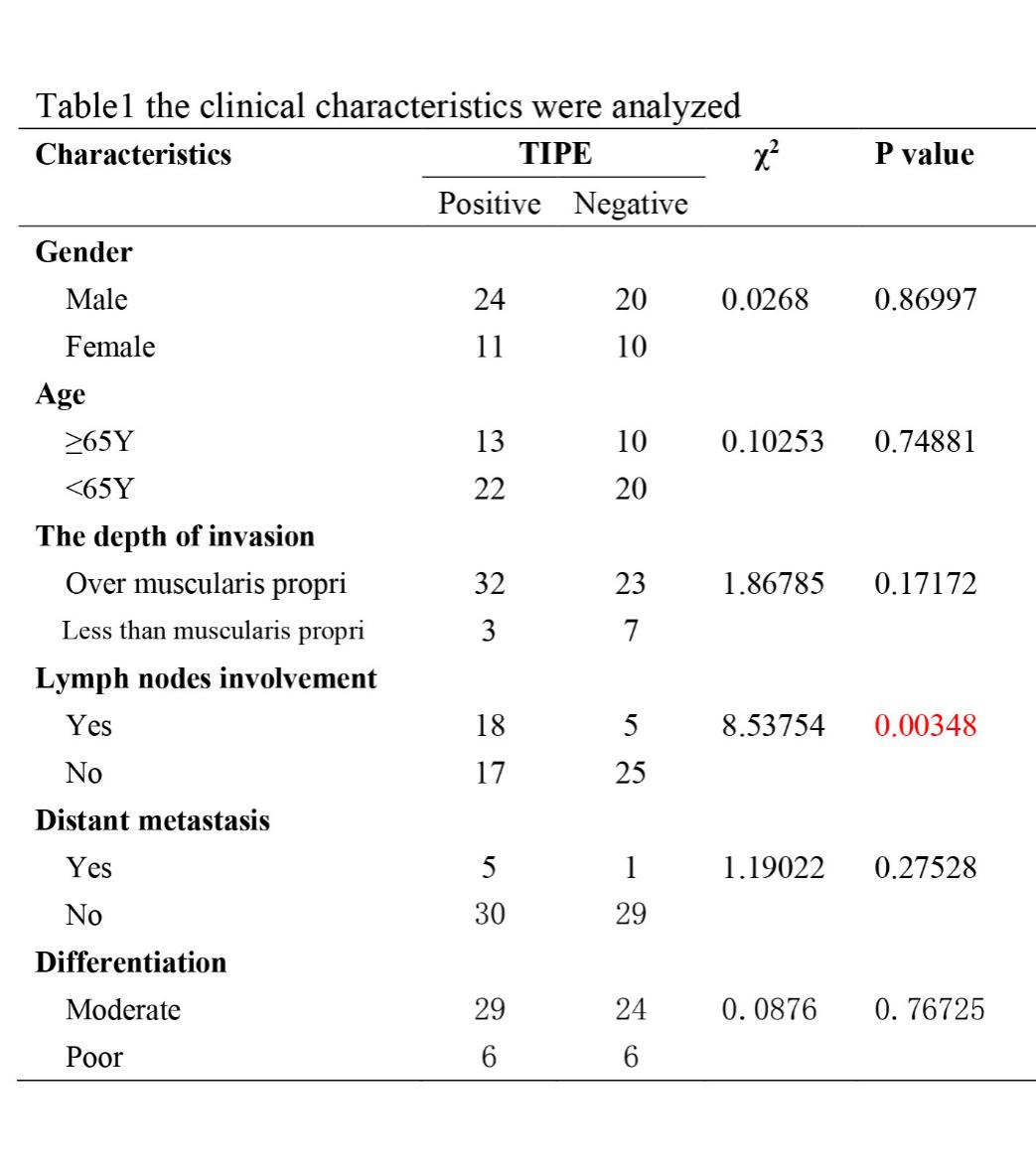


**Table 1.** The relationship between TIPE and clinicopathological factors of CRC patients

**MATERIALS AND METHODS**

**Cell culture**

Human colorectal cancer cells (HCT116, SW480, SW620 and HT29) and human embryonic kidney (HEK) 293T cells were provided from Cancer Research Center of Xiamen University (XMU, Xiamen, Fujian, China). All cells were identified by STR profiling based on the cell bank. All of the cells except HCT116 cells were cultured in Dulbecco's modified Eagles medium (DMEM, Hyclone, Logan, UT, USA) contained heat-inactivated 10% fetal bovine serum (FBS, Gibco, Waltham, MA, USA), 100 U/ml penicillin and 0.1 mg/ml streptomycin (Invitrogen, Waltham, MA, USA). HCT116 cells was maintained in 90% of McCoy's 5A (Thermo Fisher Scientific Inc, Waltham, MA, USA) contained heat-inactivated 10% fetal bovine serum (FBS, Gibco), 100 U/ml penicillin and 0.1 mg/ml streptomycin (Invitrogen). The cells were cultured in an incubator at 37°C with 5% CO_2_.

**Transfection**

A lentiviral system was used to establish the stably transfected cells. Lentiviral vector (pSIREN-RetroQ) was gifted from the laboratory of Professor Jin Guanghui, School of Medicine, XMU. The vector encoding the indicated interference fragment of TIPE were constructed and verified. Retrovirus vector (PLNCX-2) containing *TIPE* cDNA was also constructed and tested. TIPE or shTIPE plasmid and packaging vector were added to 293T cells according to the manufacturers' instructions (jet PRIME, Polyplus, New York, USA) to prepare retroviruses. The virus supernatant was harvested and centrifuged at 1000 rpm for 3 minutes to remove the cell debris. Forty-eight hours after transfection, the logarithmic phase of CRC cells were transferred into a 6-well plate (60% of the confluency). CRC cells stably overexpressing TIPE were selected by exposing to 600μg/ml of G418 (Thermo Fisher Scientific Inc). The stable shTIPE cells were selected by exposing to 6 ng/ml of puromycin (Sigma-Aldrich, St. Louis, MO, USA). These cells were cultured in a continuous screening medium for seven days, and then the concentrations of the drugs were reduced (300 μg of G418, 3 ng of puromycin) followed by the additional two weeks of the incubation. The expression of TIPE in the indicating cells was confirmed by qPCR and Western blotting.

**Quantitative real-time PCR (qRT-PCR)**

All of human CRC clinical specimens (65 patients) were obtained from Gastrointestinal Surgery Department of Zhongshan Hospital affiliated to XMU. The ethics committee approved all of the clinical samples of Zhongshan Hospital after obtaining informed consent. Total RNA was isolated from the tissues and cells by TRIzol reagent (Transgen, Beijing, China) under the manufacturer's instructions. One microgram of RNA was used as a template and reverse-transcribed to cDNA by All-in-one First-Stand cDNA Synthesis SuperMix for qPCR kit (Transgen) according to the manufacturer's protocols. Quantitative real-time PCR (qRT-PCR) reaction was performed using All-in-one First-Stand cDNA Synthesis SuperMix for qPCR kit (Transgen) under the protocol, and data acquisition was enforced on an Applied Biosystems real-time PCR System (Life Technologies, Hercules, California, USA). The primer sequences were as follows, *TIPE*-F: TTCAGGCCTCCCTCTTTAACAATC; *TIPE*-R: CGTTCGTGGCAGGGGTTATT; *MMP-9*-F: GTCCACCCTTGTGCTCTTCC; *MMP-9*-R: CTATCCAGCTCACCGGTCTC; *ACTIN*-F: AGCGAGCATCCCCCAAAGTT; *ACTIN*-R: GGGCACGAAGGCTCATCATT. The relative gene expression levels were determined using *β-actin* as the control by the formula: 2^−ΔΔCt^ [ΔΔCt=ΔCt (targeting gene)- ΔCt (control)].

**Transwell assay**

Transwell plates coated with Matrigel (Corning, NY, USA) with a 6.5 mm diameter filter and 8.0 μm pore size were used to detect cell migration capacity. Cells (8 x 10^4^) were seeded in the upper compartment of Transwell chamber, and 200 μl of the serum-free medium was injected. Six hundred microliter of 20% FBS-containing medium was added to the lower 24-well plate. Transwell plates were incubated in a standard environment to allow cells to enter the lower well from the upper chamber. After maintaining for 24 or 48 hours, the surface cells were removed by a wet cotton swab. The cells were fixed with 4% paraformaldehyde for 10 min, and then stained with crystal violet for 3 min. Number of the invading cells in the randomly selected five independent fields was scored.

**Immunoprecipitation and mass spectra analysis**

293T cells were transfected with the expression plasmid for Flag-TIPE. Forty-eight hours after transfection, transfected cells were lysed in a lysis buffer (50 mM Tris-HCl (pH 7.4), 150 mM NaCl, 10% glycerol, 1 mM EDTA) and centrifuged at 3000 rpm to remove cell debris. Whole cell lysates were incubated with protein A/G (Cell Signaling, Danvers, MA, USA) magnetic beads. The reaction mixtures were centrifuged and the supernatant was mixed with Flag M2 protein A/G magnetic beads overnight. The reaction mixtures were then centrifuged to remove the supernatant. The magnetic beads were extensively washed with lysis buffer, mixed with 2 x loading buffer and boiled for 20 min. After the denaturation, the samples were subjected to electrophoresis. After the electrophoresis, the gel strip was cut out with a clean blade and washed thoroughly with water. Then the gel was cut out into 1.5 mm x 1.5 mm blocks and put into the 1.5 ml centrifuge tube. Then, the gel was stored in 1 mL of pure water and processed for the mass spectrometer for analysis.

**Co-immunoprecipitation (Co-IP) assay**

293T cells were simultaneously transfected with the expression plasmids for Flag-TIPE and HA-MEK3. Forty-eight hours after transfection, cells were lysed in 400 μl of lysis buffer containing a protease inhibitor mixture (Sigma-Aldrich). Whole cell lysates were mixed with 20 μl protein A/G beads (Santa Cruz, CA, USA) and 1μg HA-tag antibody, and Incubated at 4 ° C for 4 h. After the incubation, the reaction mixtures were centrifuged and the resultant beads were washed four times with lysis buffer. The beads were mixed with 2 x loading buffer boiled for 20 min, and analyzed by Western blotting.

**Western blot analysis**

The indicated cells were lysed in RIPA buffer (Sigma–Aldrich) containing 1% of protease inhibitor cocktail and 1% of Phenylmethanesulfonylfluoride fluoride (solarbio, Beijing, China) at 4 °C. The protein concentration was determined by BCA Protein Assay Kit (Thermo Fisher Scientific Inc), and the equal amounts of cell lysates (20-40 µg of proteins) were subjected to 12% SDS-PAGE. After electrophoresis, proteins were transferred onto PVDF membrane (Millipore, Billerica, MA, USA), and then the membranes were blocked with 5% FBS at room temperature for 1 h, followed by the incubation with rabbit monoclonal anti-TIPE (1: 2000; Abcam, Cambridge, MA,USA), rabbit polyclonal anti-MMP-9 (1: 1000; Affinity, Changzhou, China ), rabbit monoclonal anti-MKK-3 (1: 1000; Cell Signaling), anti-P-MKK-3 (1: 500; Cell Signaling), anti-p38 (1: 1000; Cell Signaling), anti-P-p38 (1: 1000; Cell Signaling), anti-NF-kB p65 (1: 1000; Cell Signaling), anti-NF-kB P-p65 (1: 1000; Cell Signaling), or with anti-β-actin (1:1000; ZSGB-Bio, Beijing, China) antibody at 4 °C overnight. After the incubation with the primary antibodies, the membranes were rinsed and probed with horseradish peroxidase (HRP)-conjugated goat anti-mouse IgG, or with HRP-conjugated goat anti-rabbit IgG (1:2000; ZSGB-Bio) for 1 h at room temperature. Then, the immune-reactive bands were detected by Bio-Rad ChemiDoc XRS+ Detection System (Bio-Rad, Hercules, CA, USA).

**MEK kinase-3 (MKK-3) and p38 inhibition assay**

The indicated cells were treated with TNF-α (10 ng/ml, Sigma). At the indicated time points after the treatment (0, 10, 30 and 60 min), whole cell lysates were prepared and protein concentrations were determined. Whole cell lysates were analyzed for MKK-3, P-MKK-3, p38, P-p38, NF-kB p65 and NF-kB P-p65 by Western blotting. For siRNA-mediated silencing, CRC cells were transfected with 2 μl of *MKK-3* siRNA（sc-156010, Santa Cruz）and MKK-3 siCtrl. Twenty-four hours after transfection, cells were exposed to TNF-α. For inhibition of p38, CRC cells were treated with p38 MAPK inhibitor SB203580 (20 µmol/l; MCE, Monmouth Junction, NJ, USA). Two hours after the treatment, cells were incubated in the presence of TNF-α or DMSO. The phosphorylation levels of p38 and NF-kB p65 were detected by Western blotting.

**Lung metastasis model**

Lung metastasis model was established by tail vein injection. The nude mice were purchased from Shanghai SLAC Laboratory Animal Technology Co., Ltd. and placed in the Animal Center Laboratory of Xiamen University. The animal regulations have been pre-approved by the Institutional Animal Care and Use Committee of Xiamen University, and all experiments comply with the management standards involved. Twelve male BALB / c nude mice (4-6 weeks old) were randomly divided into the shTIPE group and the control group. 5×10^6^ cells suspended with 100ul PBS and injected into nude mice by tail vein injection within 5 seconds. The nude Mice were observed after injection and they were kept at specific pathogen-free condition. Forty-five days later, the mice were sacrificed to remove lung tissues. Half of the lung tissues was fixed in formalin for pathological H & E staining, and the remaining lung tissue was extracted as RNA and protein for qRT-PCR and Western Blot detection.

**H&E staining**

All tissues were fixed with 10% formalin, dehydrated routinely, embedded in paraffin and sections were 5 μm in thick. Dyeing with hematoxylin and eosin.

**Statistical analysis**

The experimental data were analyzed by software SPSS version 15. The densitometric quantification of cell numbers was made using ImageJ software. Quantity One quantified protein and RNA bands. We use the paired t-test, or one-way factorial ANOVA to evaluate the significance of the difference between groups, and the data were presented as the（mean ± s.e.m in three separate experiments）. p<0.05 was used to indicate a statistically significant difference.
