# Supplementary figures and images for: TIPE-mediated up-regulation of MMP-9 promotes colorectal cancer invasion and metastasis through MKK-3/p38/NF-κB pro-oncogenic signaling pathway
Source: Signal Transduct Target Ther. 2020 Aug 25;5:163. doi: 10.1038/s41392-020-00276-7 (PMC7447793; doi:10.1038/s41392-020-00276-7)

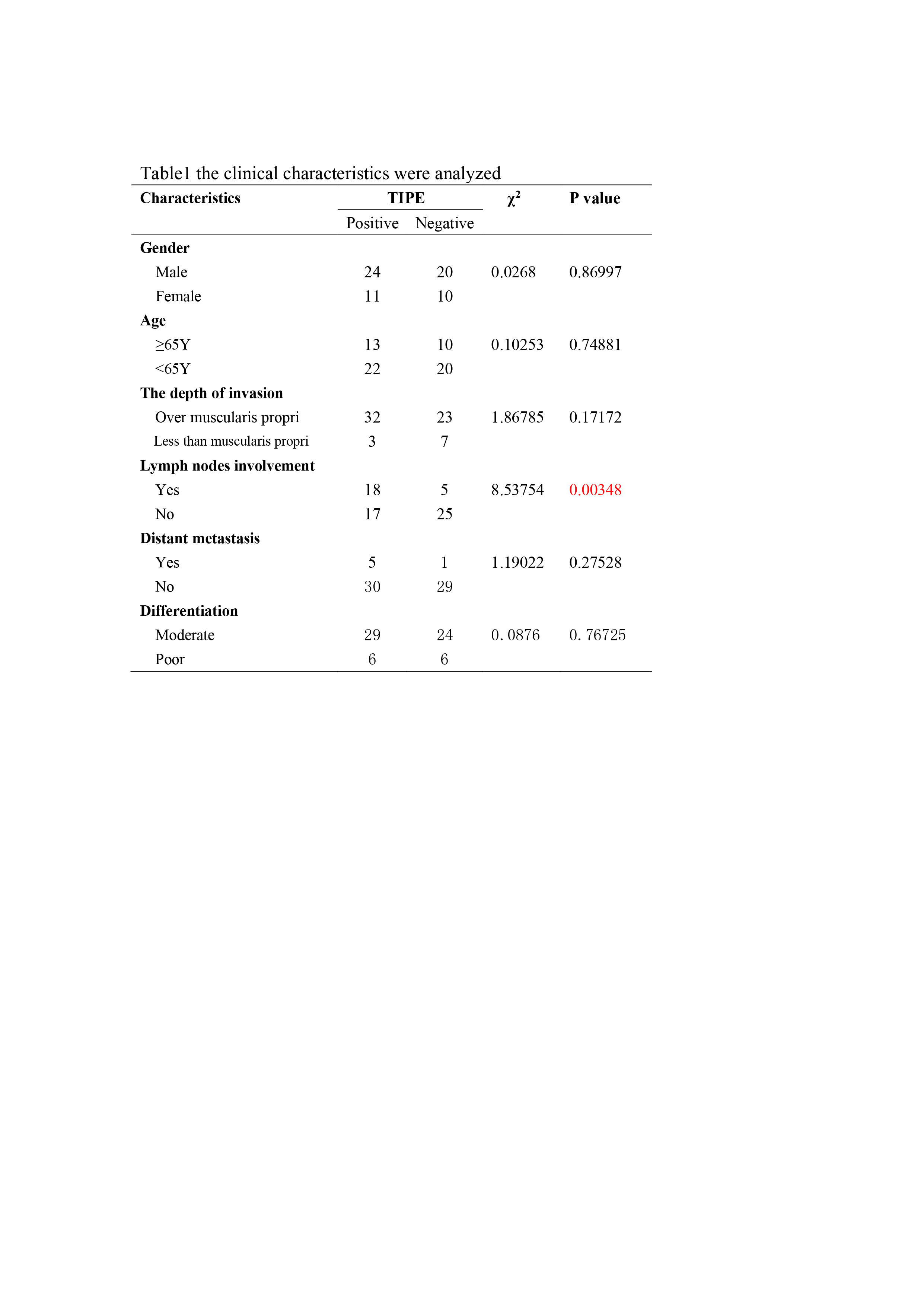

Supplement: Supplementary file 2 — Supplementary Table 1. The relationship between TIPE and clinicopathological factors of CRC patients [file 41392_2020_276_MOESM2_ESM.jpg]
